# Meditation as a Potential Therapy for Autism: A Review

**DOI:** 10.1155/2012/835847

**Published:** 2012-06-04

**Authors:** Sonia Sequeira, Mahiuddin Ahmed

**Affiliations:** ^1^Office of Clinical Research, Memorial Sloan-Kettering Cancer Center, 1275 York Avenue, New York, NY 10065, USA; ^2^Naam Biomedical Society, 228 Park Avenue S21210, New York, NY 10003, USA; ^3^Department of Pediatrics, Memorial Sloan-Kettering Cancer Center, 1275 York Avenue, New York, NY 10065, USA

## Abstract

Autism is a chronic neurodevelopmental disorder of unknown cause that affects approximately 1–3 percent of children and four times more boys than girls. Its prevalence is global and its social impact is devastating. In autism, the brain is unable to process sensory information normally. Instead, simple stimuli from the outside world are experienced as overwhelmingly intense and strain the emotional centers of the brain. A stress response to the incoming information is initiated that destabilizes cognitive networks and short-circuits adequate behavioral output. As a result, the child is unable to respond adequately to stimulation and initiate social behavior towards family, friends, and peers. In addition, these children typically face immune-digestive disorders that heighten social fears, anxieties, and internal conflicts. While it is critical to treat the physical symptoms, it is equally vital to offer an evidence-based holistic solution that harmonizes both their emotional and physical well-being as they move from childhood into adult life. Here, we summarize evidence from clinical studies and neuroscience research that suggests that an approach built on yogic principles and meditative tools is worth pursuing. Desired outcomes include relief of clinical symptoms of the disease, greater relaxation, and facilitated expression of feelings and skills, as well as improved family and social quality of life.

## 1. Background

Autism belongs to a group of related disorders that starts in infancy and remains throughout adult life [1]. Impaired social interaction at a young age affects early milestones of human development and a myriad of immune deficiencies will also afflict the majority of these children [2–8] The etiology of autism is not known. However, it is currently agreed that a combination of genetic and epigenetic factors, contribute to autism, the first trimester of pregnancy being a particularly vulnerable time to triggers of the disease. Autism is characterized by widespread disruption of the brain networks that underlie complex cognitive and emotional functions that results in an imbalanced neurological response to cues from the external world and, particularly, in the way the child responds to stress. 

A myriad of treatments for autism have been proposed; however, in most cases the existing data are insufficient to support their efficacy [9]. This is primarily due to the confounding complexity of genetic traits of the disease, as well as the difficulty in distinguishing the cause from downstream pathologies. And yet in the United States, the lifetime cost of care for an individual with autism can reach as much as $3.2 million [10].

## 2. Social Interaction

Social interaction from an early age is fundamental to human development at many levels. The development of skills, relationships, and character depends on adequate and repeated brain stimulation to enforce strong networks that support decision making for well-being [11]. Furthermore, it is necessary to protect the brain from unhealthy sensory overload [12]. Maintaining a healthy balance between receptivity and detachment to others is critical to flourishing relationships and to physical and mental health. Lifestyles that include regular exercise and meditation have been shown to have a greater chance at achieving such equanimity [13]. Infants and children have signature brainwave patterns that influence physiological rhythms and mental capacity adequate to their stage of development. From newborn to two years of age, a predominantly delta (0.5–3.5 Hz) wave pattern supports a strong immune system, anesthesia, release of growth hormone and, limited awareness of the physical world [14]. From the age of 2 to the age of 6, brainwaves shift into the theta range (4–7 Hz) where creativity, learning and, a high tolerance to stress maximize the child's success in creating social skills. Although much is yet to be learned in this field, research has clearly shown that synchronicity of these events and of neuronal activity *per se* is a critical part of higher cognitive function, perceptions, and behavior [15–17]. At this tender age, symptoms and abnormal physiology begin to emerge in persons with autism consistent with the progression of inadequate neuronal development and abnormal brain synchronization [18–20]. Lack of brain synchronicity may be responsible for the atypical sleep and stress mechanisms in these children [19] and changes in brain activity signatures may be predictive of disease and its severity. The inability to regulate social interaction through basic behaviors such as eye contact, facial expression, and body gestures is common in children with autism [1]. These children typically lack spontaneity to share enjoyment and achievements with others. This becomes a major hindrance and frustration to the development of peer and child-teacher relationships in the school setting [9, 21, 22].

Meditation, both traditional and more modern, is a tool to access higher states of consciousness. It is said that in these higher states of consciousness duality is dissolved, conflicting thoughts and feelings subside, and one regains a sense of connectedness, tranquility, and equanimity. This search for a deeper and vaster meaning to life, beyond immediate or gross desires and memories, involves finding new possibilities and greater potential to manifest a more productive and healthier life. Meditation is a stepwise process that begins with focusing on an object associated with positive, higher principles (such as compassion). As the practice continues, the focus naturally shifts from the physical object itself to the meaning of the object and, eventually, objectless concentration is achieved, or merger of observer and observed. When monitored by clinical EEG, the meditative process is accompanied by distinctive stages of brain activity and results in greater synchronicity of brain patterns in both new or long-term meditators (for review see [23]). Several physiological changes associated with these brain patterns occur, in particular ones that support the immune system [24–28]. The significance of meditation as a strategy to treat chronic neurological diseases is not confirmed by evidence-based research. However, it has been shown that a variety of forms of meditation have been beneficial adjunct therapies in anxiety [29–32], depression [33–35], epilepsy [36], posttraumatic stress disorder [37], and pain [34, 35, 38–40]. Meditation is described in great detail in the Vedas (approx 1500 BC), the oldest written texts found to date, and in a succession of commentaries and corollaries over time, such as the Upanishads (pre-Buddhist period to 500 BC) and the Yoga Sutras (100 BC to 500 AD) [41–43], that interpret the meaning and distill the key sounds of the Vedas (mantra), such as AUM (OM).

As early as 700 BC, universities in the Indian Subcontinent included Physiology and the Vedas in their medical syllabus and attracted students from as far as Babylonia, Greece, Syria, Arabia, and China. Higher education was therefore a major vehicle of dissemination of meditation to the West. Similarly, Buddha brought meditation to the Himalayan plateau from India. Migration and trade between the Indian Subcontinent and the Fertile Crescent later influenced Egyptian, Sufi and Christian-Judaic meditation. This influence is still traceable to this day in Jewish and Gregorian chanting traditions. However, the vedic texts were only recently compiled and translated, causing a renewed interest and study of the methodology of meditation [44–46]. Vedas are songs or hymns intended to be recited for physical and spiritual well-being and progress, of both the individual and society. Many methods of meditation have been derived from the original Vedas, and it is important to scientifically weigh their therapeutic value, in face of their antiquity and benign nature [36].

For the purposes of the present paper, which focuses on autism, it is particularly important to systematically study these different forms given that the youngest children affected by this condition are not yet able to sit still and direct attention. In these children, the use of mantra (singing) will be a particularly interesting hypothesis to test. Programs for autistic children that include mantra have been previously reported [47] and in the authors' own experience, mantra is a feasible and beneficial practice in a clinical setting with children as young as 3 years of age (data in preparation for publication). It is also well known that the human brain can be entrained and synchronized by musical stimuli—especially in very young children [48].

In this review, we propose that meditation practices receive more attention in the scientific and policy-making arena as a potential intervention to treat or relieve symptoms of autism. 

## 3. Imitation

The neural basis of the human ability to read minds and predict other peoples' intentions is a specialized network of neurons in the frontal cortex called the “mirror system” [49]. Mirror neurons are active when one performs a certain task but also when one observes the same task performed by others. In other words, the brain forms a theory of the other's mind using one's own experience. This mechanism enables imitation learning and explains the feeling of empathy towards other beings. Having emerged early in the evolutionary development of the human brain, this system also plays a critical role in the formation of language and cultural inheritance. Imitation is an early milestone in child development, and impaired socialization and lack of imitative play in children with autism are among the diagnostic criteria for the disability. Indeed, in autism, this network is underdeveloped, resulting in deficient copying skills and the inability to recognize and interpret another's mind [49–54]. Noninvasive interventions using rhythm, such as dance and singing, present great potential in the treatment of autism because rhythm inherently entrains movement through the stimulatory effect on residual mirror neuron function, and children are most receptive to these cues [47, 55].

## 4. Empathy

By the age of two, children normally begin to display the fundamental behaviors of empathy. Empathy is an emotional response that begins with the recognition of another person's mental and emotional existence. Awareness that others' mental position may be different than one's own is called the “theory of mind,” coined by Humphrey and Baron-Cohen [56]. In the theory of mind, one evaluates other's emotions in reference to their own emotions instead of simply picking up others' emotions [49]. This process of adopting others' mental state and evaluating it from one's own perspective, anticipating intentions and feelings, is essential for social interaction and a stepping stone to the virtue of compassion. Several studies indicate that the theory of mind may be disrupted in autism, involving the mirror system of neurons and its connections to emotional and associative centers of the brain that construct consciousness [57–59]. In essence, the child with autism is unable to create self-consciousness and as a result consciousness of others. For this reason, the exploration of noninvasive meditation techniques that foster self-awareness and feelings for others, as an extension of self (higher consciousness), is a natural direction for autism research. Given its benign nature and ability to protect and shape the brain, as well as its solid research record in other neurological conditions [29, 34, 36, 39, 40, 60], there have been surprisingly few reports of its use for autism. Meditation is unlikely to present itself as challenging to these children and individuals, if built upon a yoga practice that includes movement, breathing, and chanting [47]. Moreover, this form would be easily read and interpreted by investigators.

## 5. Molecular Mechanisms of Autism

The brain is composed of three distinct tissue types: gray matter, white matter, and cerebrospinal fluid. Grey matter is composed of neuronal cell bodies, dendritic extensions, and supporting glial cells. These are the structures involved in synaptic transmission, the basic process that underlies memory. White matter corresponds to the long, fat-insulated axons that carry information to distant neurons. Measurement of the thickness and surface area of these tissues provides information on the density, number, and degree of myelination of these neurons, respectively. Being highly plastic, brain tissues undergo short- and long-term changes in structure when cognitive tasks are performed and repeated. These changes have been associated with cognitive performance in studies that show that individuals who score highest in general intelligence in standardized tests have thicker grey and white matter in specialized areas [61]. On the other hand, F-18 fluorodeoxyglucose positron emission tomography (FDG-PET) has shown that brain glucose consumption is lower in more intelligent individuals, suggesting that rather than just growth, these changes reflect the fact that neurons are used more efficiently. It is therefore hypothesized that neuronal activity causes network “pruning” or the formation of new synapses and concurrent elimination of old synapses, resulting in a positive increase in synaptic efficiency, tissue thickness, and enhanced cognitive ability [62, 63]. Importantly, greater cognitive performance does not correspond to global enlargement of the brain but instead reflects changes in short- and long-distance wiring resulting in localized growth.

In autism, several structural abnormalities of the brain have been reported. Particularly striking is the abnormal overgrowth of the brain cortex surface area (grey matter) and the thickening of white matter in the corpus callosum and temporal lobe in autistic children before the age of two years [20, 64]. This initial phase of brain enlargement is followed, in adolescence and young adulthood, by arrested growth and accelerated thinning of the cortex in areas involved in cognitive performance and the formation of emotional memory [65, 66]. The observed bulking seems to indicate aberrant migration of neurons, a greater number of long-distance neuronal projections, or excessive myelination that inadequately wire the brain. It is possible that these changes amplify sensorial processing and the ability of the brain to retain and “feel” auditory and visual experiences, as also evidenced by the similar pattern of early growth and later regression is shown in the amygdala, the structure responsible for adding emotional memory to experiences [22, 57, 67]. Similar changes in convolution have been observed in other pathologies such as attention deficit hyperactivity disorder (ADHD) [68] and Parkinson's disease [69]. Among the areas where cortical thinning is observed in adults with autism is the mirror neuron system (inferior frontal gyrus) where the degree of thinning is proportional to the severity of symptoms experienced [52]. The mirror neuron system, as mentioned previously, is essential for learning imitation and the neural correlate of empathy [49, 54]. It seems, therefore, that early events in brain development disrupt the structural organization of the fully developed adult brain, affecting in particular the connections between the temporal, parietal, and frontal mirror systems that jointly formulate the theory of mind and the basis of social reciprocity [50, 59, 70]. In parallel to these structural changes, individuals with autism show atypical activity in these areas and significantly decreased connectivity across their functional networks [54, 71]. Together these studies suggest that the social and emotional deficiencies in autism are associated with cortical thinning of the functional networks supporting social interactions, possibly due to errors in neuronal migration or apoptosis in early development [52, 72]. Interhemispheric transmission of information is also compromised by the thinning of the corpus callosum (CC), and, as a result, also the capacity to integrate complementary experiences such as musicality and language, mathematical relations, and abstract symbols [64, 73]. The CC is a white matter structure and the largest commissure in the human brain, connecting the right and left hemispheres by more than 200 million fibers. A larger CC is associated with better cognitive performance, perhaps because additional or better-myelinated callosal pathways facilitate a more efficient interhemispheric information transfer likely to benefit the integration and processing of information and positively affect intellectual performance [74].

Disrupted neuronal axon function might explain why, in autism, long-distance connectivity of different areas of the brain involved in emotion processing is impaired, but also why it may be difficult for these individuals to shift attention from a particular task to another, resulting in repetitive behavior [75–77]. In this scenario, long-distance under-connectivity caused by a decrease in the number of large axons would explain the impaired coordination and integration of time-sensitive information necessary from different brain regions, resulting in deficient social interaction and language skills. On the other hand, local overconnectivity associated with an excess of locally communicating short axons can be linked to the repetitive and restrictive behaviors observed in autism [22]. Hyperfunctional local neuronal microcircuits, with excessively reactive neurons and synaptic plasticity, are the likely cause for the characteristic hyper-perception, hyperattention, and intense emotional response to stimuli (such as light, touch, and sound) in children with autism [78–80]. Indeed, the prefrontal and somatosensory cortexes, and the amygdala, are significantly more active and plastic when a stimulus is presented [81, 82]. The excess amount and intensity of information flowing through the amygdala is likely to cause a severe stress-response and emotional overload, with heightened experiences of fear and anxiety that culminate in self-withdrawal and decreased social interaction. This mechanism is consistent with the observation that children with autism have greater levels of stress hormones in their blood and heightened autonomic nervous system activity [83, 84] and why anxiety and phobias are frequent comorbidities of autism [21]. Also in the somatosensory cortex, exacerbated sensory representations of the body are transferred to areas of integration that are unable to properly process the information and carry out behaviors.

At the molecular level, some suggestions have been made to explain the functional and structural changes in autism described above. Inhibitory neurons, which control excitability and reduce the duration of each stimulus, may undergo abnormal cell death in these functional areas resulting in more frequent and more intense activity. In the cerebellum, for example, a greatly reduced number of inhibitory Purkinje cells is observed [67]. Overexpression or irregular function of NMDA receptors of glutamatergic circuits in particular pyramidal neurons of the cortex, which are highly plastic or adaptive to stimulation [22, 82, 85, 86], and their associated transduction pathways may also be responsible for these effects by increasing postsynaptic excitation [87, 88]. Genetic studies have identified synapse gene mutations on chromosome 22 [89–92] and are therefore consistent with a model of heightened synaptic plasticity in autism.

In summary, a molecular sensitization of yet unknown origin lessens the protective functions of the brain, leading to an extreme response (hyperplasticity, hyperperception, hypermemory, hyperattention, and hyperemotionality) to external stimuli that would otherwise provide an enriching environment for brain development. At a young age, strong reactions and memory of experiences in subcortical connections may prevent or outcompete normal development of long-distant connections, higher order cognition.

## 6. Meditation

After more than 20 years of committed research in autism, there is ample reason to pursue alternative solutions to treatment. Prospective studies have generally found that 60–75 percent of individuals with autism followed into adulthood experience poor or very poor outcomes [1]. There is also no effective medication for autism. Risperidone, the only FDA-approved drug for the treatment of altered behaviors in autism, has known adverse effects and is only indicated for moderate-to-severe behavioral problems associated with autism since there is no clear benefit in treating core symptoms. The economic burden of this disease to affected families and indirectly to society is unbearable, reaching $3.2 million per individual in the USA. The current model of care is therefore unviable to transfer to developing countries where the prevalence of autism is equally high. Because autism is a pediatric neurodevelopmental disease carried into adulthood, the search for nondrug alternatives to treatment is warranted. Several approaches have been suggested such as acupuncture, massage, auditory integration training, detoxification, and neurofeedback. However the studies available so far are insufficient to support or oppose their validity [93].

Meditation is a conscious process of self-regulation that tempers the flow of thoughts, emotions, and automatic behaviors in the body and mind. In our accomplishment-driven society, the human brain constantly receives and processes countless pieces of information from the outside world that are contradictory, opposing, and threatening to the organism. As a result, the brain commands a stress response in the body that recruits defense mechanisms and demands high expenditure of energy resources that can severely tax the body in the long run. It is now recognized that cognitive stress is linked to accelerated cellular aging and DNA degeneration [25, 94] that can span generations [95–98]. Furthermore, research suggests that practically every chronic disease of the western world is caused or triggered by stress, particularly in early life [27, 99–103].

 As one of the earliest recorded systematic approaches to promote health and longevity, the unifying principle of meditation-centered disciplines that have emerged over thousands of years is to move from a state of duality, characterized by conflict or maladaptation to one's environment, to one of singularity or harmony. Singularity corresponds to a psychological state of unity, timelessness, and totality in which threat cannot exist, since there is nothing exterior or beyond our being-universe continuum. From a physicochemical standpoint, relaxation in the present moment is the state of maximum preservation of our energy resources, harnessed for optimal health and inspiration towards higher ideals.

Yogis and eastern physicians knew of the existence of the circulatory and endocrine systems and circadian rhythms as early as the 6th century BC [46, 104, 105] and describe a more refined level of signaling in organisms at the physical and atomic level than the chemical and molecular known to modern medicine today [42, 46, 106]. (e.g, verses 1–12 of the Mandukya Upanishad describes four levels of consciousness from waking to pure consciousness, and nineteen channels: four functions of mind, which are manas, chitta, ahamkara, and buddhi, that operate through five pranas (prana, apana, samana, udana, and vyana), the five active senses and the five cognitive senses.) Rather than ignoring this vast amount of information and the millenary arts from it derived, it is naturally desirable to yield these principles to scientific rigor to understand them.

Meditation creates a one-pointed mind that helps restore the coherence of the human system and as well as harness energy to overcome physical and psychological challenges. As a complementary approach to personalized medical care and conventional therapies, meditation has shyly made its way into several clinical settings and has undergone unprecedented scientific research in the last few years. The main driving force for this sudden interest is the undisputed need to develop less costly health programs for rising chronic diseases and noninvasive, self-initiated strategies to promote wellness in a fast-paced, technology-dependent society that promotes multitasking, constant sensorial stimulation, and less contact with Nature [12, 107–110].

Moreover, neurological disorders that severely impair social integration, professional development, and quality of life have found no solution in drugs or clinician-facilitated psychology [111, 112]. Discovery or rediscovery of mental training strategies to protect the human brain while stimulating the mind and better process our perceptions of the world is therefore of great interest. And most importantly, a great deal of research is necessary for us to learn how to use these tools in younger populations [108, 113–115].

Traditional yogic exercises and meditation techniques constitute a comprehensive body of knowledge that shows a profound and thorough understanding of human physiology and neuroscience obtained through experimentation and systematic practice that rivals that established by the western world in the last century [42, 45, 104, 116]. Through our modern advances in imaging and nanotechnology, we seem now to begin to understand the molecular underpinnings and objectives of each component of these ancient practices. To facilitate the study of the neural correlates of meditation practices from different traditions (Chinese, Indian, and Buddhist), scientists have divided them into three main categories of cognitive processes reported by brain activity [23, 117, 118]: those that emphasize concentration or focused attention on an object [119, 120]; those that emphasize moment-to-moment, dispassionate observation of experiences [117, 121]; those that transcend the object of focus automatically [23, 118]. Each of these cognitive processes (focus, nonjudgmental observation, and effortless transcendence of practice) is associated with a signature brainwave activity recorded by electroencephalography (EEG) (for review see [23]). Focused attention on an object such as feelings of compassion during meditation has been shown to elicit gamma (30–50 Hz) and high-range beta activity (20–30 Hz) as well as increased frontal-parietal gamma coherence and power [122]. Practices that emphasize non-judgemental focus on the flow of thoughts and experience such as Vipassana or Sahaja Yoga have shown a predominantly theta activity in the frontal midline cortex (5–8 Hz) [120]. Lastly, practices that focus on transcending the object of meditation such as Transcendental Meditation have been shown to elicit frontal alpha activity (8–10 Hz) [123]. Each characteristic brainwave is in turn associated with specific physiological changes such as increased immunity, regeneration and growth, deep sleep, and high concentrative power. “Tuning-in” to specific brainwaves regularly through meditation seems to shift brain activity to states of greater coherence, power and plasticity or task-appropriate frequencies. States of heightened alpha brainwave, considered the gateway to meditation states, promote physical relaxation and cognitive performance, and regular training may facilitate day-to-day troubleshooting and socialization [121].

This classification is a notable initial attempt to organize meditation into meaningful groups but it is important to keep in mind that duration and repetition of the practice are factors that will almost certainly change the patterns of brain activity. Furthermore, there are uncharted forms of traditional meditation that have not been included in this classification, such as those that involve chanting of mantra accompanied by mudras or hand gestures that facilitate focused attention and interhemispheric synchronicity set into motion by rhythmic vocal sounds and breath patterns. This older form of meditation is inherently easier to adhere to and to monitor. Recently, it has become known to western practitioners through disciplines such as Kundalini Yoga as taught by Yogi Bhajan and Naam Yoga [47, 60, 124, 125].

Breathing exercises are part of the meditative process and are often an object of focus but are particularly interesting in the context of autism given that most children with this condition have left nasal dominance [126]. Yogic breathing includes exercises that force the flow of air through the right or left nostril alternately that result in selective activation of the sympathetic or parasympathetic branches of the autonomic nervous system [127]. Left nostril breathing while closing the right nostril has a calming effect and has been shown to increase vagal tone and right hemisphere dominance while right nostril breathing is associated with increased sympathetic and left hemisphere activity [30, 127]. Alternate nostril breathing patterns may help correct abnormal breathing patterns and physiological rhythms. In general, breathing control exercises autonomic balance, hemispheric performance and mood, and both meditation and yogic breathing have been suggested to induce the secretion of estrogenic hormones such as oxytocin that facilitate bonding and affection [127, 128]. It has been postulated that a prenatal or neonatal exposure to excess testosterone may explain the fact that autism is much more frequent in boys than in girls, who are less vulnerable due to the protective effect of oxytocin [129, 130]. These observations suggest a beneficial role for meditation to mothers during pregnancy and postpartum periods. It is also possible that meditation in young children, particularly boys, may reduce symptoms of autism by increasing the release of oxytocin in the brain [131].

 It has been shown that diverse activities such as computerized training support executive control in early childhood are critical for success throughout life. However, it is evident that executive function development is best achieved by simultaneously addressing emotional, social, and physical development [13]. In situations of high executive demand, a combination of exercise and character building or mindfulness positively affect core executive functions such as self-control, cognitive flexibility, and working memory [13]. Successful programs do not require that children remain sitting still for very long but encourage social bonding, joy, and confidence. Focus on compassion and higher ideals in meditative practices are nurturing and can facilitate conflict resolution and socialization. Social isolation, occurring in persons with autism, may impair pre-frontal cortex mediation of executive control, once again indicating that early interventions of this nature are sorely needed. The combination of exercise, breath training, and sound designed for therapeutic use, and resembling play, may be an excellent approach to teaching meditation to children and warrants further study. Others have described benefits to teenagers with transcendental meditation and kundalini yoga [29, 132]. It is important to note that relaxation training may increase cardiac parasympathetic tone but may not confer other effects associated with meditation.

Heightened brain synchronicity is the most frequently observed effect in these practices. Structural changes in several areas of the brain have been observed in meditators [133, 134] although the correlation between these changes and improved health requires further investigation. As a matter of fact, we do not know if meditation can reverse the abnormal wiring and thinning observed in autism and other diseases, and we do not know which form of meditation would be most beneficial. However, positive subjective accounts of meditative experiences have been repeatedly documented with adults, adolescents, and even children, indicating better coping skills, less pain, improved mood, and stronger immunity [28, 35]. Furthermore, meditation improves breathing patterns, and studies showing changes in hormone levels confirm the ability for meditation to change physiological parameters and rhythms [119, 135, 136].

The practice of focus on a stationary, positive, and life-promoting symbol, whether it is breath, mantra, or an ideal, allows us to collect and silence dispersed thoughts, disharmonious feelings, and destructive behaviors that over time progressively destabilize body functions, signaling systems, and genes. In evolutionary time, stress is a primary engine for species adaptation to the environment, driving cell renewal, and gene modification that is heritable. However, in the lifetime of an individual or in a few generations, chronic stress causes several genetic mutations that rapidly accumulate in cells until they manifest into pathologies such as cancer, diabetes and arteriosclerosis [137].

During meditation, the restorative mechanisms of the body are strengthened and downregulate the uncontrolled function for survival. Life-promoting hormones such as human growth factor are secreted and more glucose is directed to the brain to fuel more efficient neuronal pathways that result in a feeling of well-being, while old habits and the neurons sustaining them “die out” [134, 135, 138].

Chanting mantra remains a part of many traditions and lineages of practice today. These patterns are used as catalysts of meditative states and Samadhi (pure consciousness) because of their perfected rhythm, sound, tone, focus, and meaning. This is not uncharted territory since it is known that musical beats have entraining power [139]. For example, it is well documented that slow-tempo musical pieces induce delta brainwaves and sleep. It is also known that language, music, and singing share functional networks and therefore singing and music may compensate for deficiencies in language [140–142]. In its original form mantra does not make use of any common language but appears to be a system of sounds or a symbolic language that, when thought of or pronounced, entrains a particular brain activity or body rhythm and creates one-pointedness in the mind. A few studies have reported these benefits; the mantra “om” has been shown to synchronize respiratory signals, cardiovascular rhythms, and cerebral blood flow while another mantra, “SaTaNaMa,” was reported to significantly change cerebral blood flow patterns measured by CT [143, 144]. Figurative translations of different mantra describe higher ideals of beauty, unity, and love and are prescribed for many ailments. However, a comprehensive comparative study of these mantra as psychophysical modulators of health has not been attempted.

## 7. Conclusion

In summary, the practice of meditation may provide unique benefits that complement a lifestyle with balanced physical exercise, good nutrition, and a nourishing environment. Meditation is one of a few interventions that have been shown to effectively strengthen self-control and character development simultaneously [13]. There are several styles of meditation available today. Each form uses different degrees of focused attention on a variety of objects to reach a clear, meditative mind. Despite the differences in methodology, they all share the objective of self-relaxation, self-healing and consequently, improved cognitive and behavioral performance. There is much to be gained by exploring meditation as a strategy to override impaired brain synchronicity and debilitating symptoms arising in early years of persons with autism. The authors suggest that mantra meditation may be most useful in young children. In a pilot program, our results indicate that mantra is a feasible intervention for children between 3 and 14 years of age to improve health outcomes, and we are implementing a clinical trial to verify our initial findings. We encourage a concerted effort from all fields of research to incorporate ages of ancient wisdom into the health challenges we face today.
